# Supplementary figures and images for: Diversity, origin, and evolution of the ESCRT systems
Source: mBio. 2024 Feb 21;15(3):e00335-24. doi: 10.1128/mbio.00335-24 (PMC10936438; doi:10.1128/mbio.00335-24)

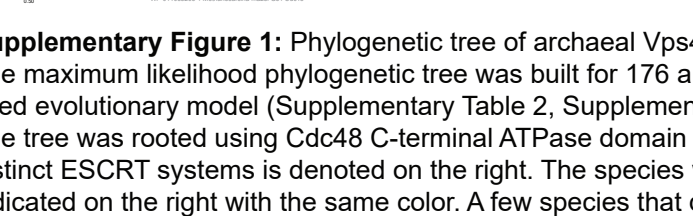

## CdvA

## Main

# Thermoplasmata

## Halo\_adaptin

## Meth\_adaptin

## Halo\_FHA

## Outgroup Cdc48

Supplement: Figure S1 — Phylogenetic tree of archaeal Vps4 tree rooted using Cdc48 as an outgroup. [file mbio.00335-24-s0001.pdf]
