# Supplementary material for: Diversity, origin, and evolution of the ESCRT systems
Source: mBio. 2024 Feb 21;15(3):e00335-24. doi: 10.1128/mbio.00335-24 (PMC10936438; doi:10.1128/mbio.00335-24)
Supplement: Figure S2 — Structural models for the main clade of ESCRT systems. [file mbio.00335-24-s0002.pdf]

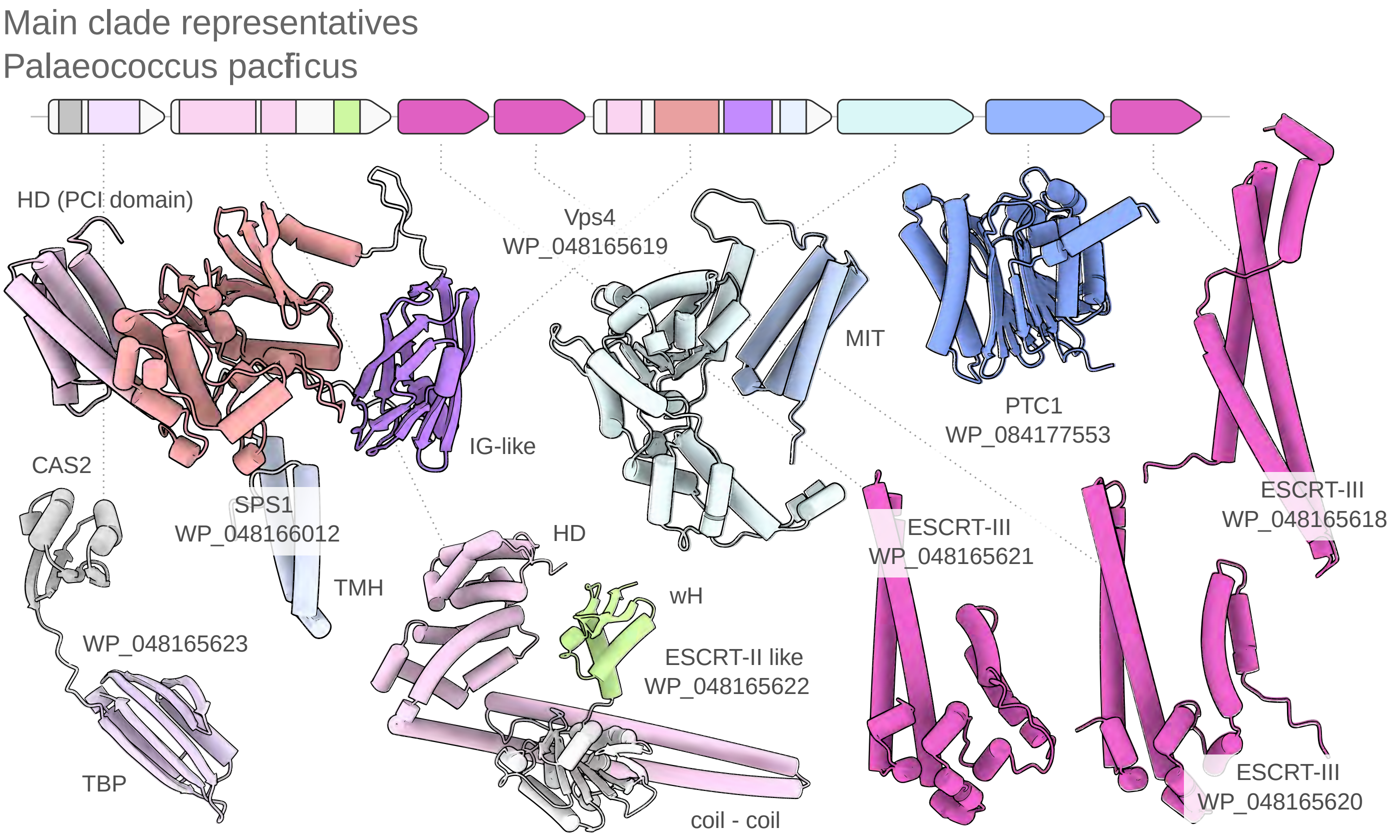

Supplementary Figure 2: Structural models for the main clade of ESCRT systems.
The gallery of structure predictions obtained for the Main clade (Figure 3A) using the gene neighborhood from Palaeococcus pacificus as representatives (Supplementary table 3) is shown. Gene neighborhood organization is shown on top. Proteins are colored by common structural domains found in ESCRT gene neighborhoods. Named proteins are assigned by sequence or structural similarity. NCBI accessions are shown for all predictions. Unstructured termini and long linkers are hidden. Abbreviation are as per previous legends, with the addition of HD, Helical Domain and TMH, Transmembrane helix.
