# Supplementary material for: Diversity, origin, and evolution of the ESCRT systems
Source: mBio. 2024 Feb 21;15(3):e00335-24. doi: 10.1128/mbio.00335-24 (PMC10936438; doi:10.1128/mbio.00335-24)
Supplement: Figure S3 — Multiple alignment of arCOG08177 sequences. [file mbio.00335-24-s0003.pdf]

```
                                 //.....................................Cas2.........................................//
WP_015313867_1.-----------------MYLVICNYDSD-----SERKRIEYVFSKSSSEADDVRKIQGITRIINDN--DIENLLSDLYLRT---SA-----HNVSVYKISK--VEVDNLP.76
OIQ05980_1.....-----------------MYLITAKYFND-----AERKRIEYIIKLWEDKV-NIIKPPGITFLVDG---KPDEIVNELASKI---SE-----KNIDVFKLDE--LSLNLEK.74
PIV28043_1.....-----------------MYLITAKYFND-----AERKRIEYIIKLWEDKV-NIIKPPGITFLVDG---KPDEIVNELASKI---SE-----KNIDVFKLDE--LSLNLEK.74
PKP60869_1.....-----------------MYLITAKYSGD-----AERKRIEYIIKLWEDKA-NIIKPTGITFFVDG---EPDEIVNDLVSKI---SE-----KNIDVFKLEK--SPLNLEK.74
ARM76973_1.....-----------------MYFLQVYYRND-----NERKRLDYIINKWNN---KVSKLDGYLLKIDDE-TTYKEIFNEISSKF---PP-----ELIKSYKAEE--LEVKPQT.74
WP_048099576_1.-----------------MYLIEVSYRNE-----QERKRLDYVLEKY--RD-STMGLEGYAFKVFDD--RYNEVASEIMRKF---PQ-----EIVKVYKVEE--VEFKEEV.73
WP_011011773_1.----------------MKYLFIVDYSDD-----AERKRIDYLLEKWQDQA-IISKPRGLIFIIETR--DINKFAEELFSKLSPQSS-----KKVRAFVIQEENLQSKIPP.81
WP_056934134_1.----------------MKYLFIVNYEDD-----AERKRIDYLLEKWQERA-RISKPRGLTFIIETD--EISKFAEELFSKLNPSSS-----KKVESFVLHEKDLSSQIRS.81
WP_048165623_1.----------------MPHLFIISYGND-----AERKRIEYLLEKWSERA-EISKLRGITFIIDTE--NVSEFAEELLSKLDPPSS-----DKVEVYHVKKEDLKSKVAP.81
WP_055429724_1.----------------MPHLFIVGYEND-----AERKRVEYLLDKWSNRA-RISKVRGISFIIDSS--DVEGFAEELLSKLDPPTE-----GKVMVYRVEPEDIGSRVSP.81
WP_062390972_1.----------------MAHLFVVVYDSD-----AERKRVEYLIDKWSNRA-GVSKLKGISFMVEAP--DVSKLMEELLSKLDPPTE-----GKVRVYRVEPEDIGSKVTP.81
WP_048152318_1.----------------MTHLFVVVYDND-----AERKRVEYLIDKWSNRA-RVSKLKGISFMVEAS--DVSKLMEELLSKLDPPTE-----GKVRVYRVEPEDIGSKVTP.81
WP_058937978_1.----------------MTHLFVVVYDSD-----AERKRVEYLIDKWSNRA-RVSKLKGISFMVEAP--DVSKLMEELLSKLDPPTE-----GKVRVYRVEPEDIGSKVSP.81
WP_015733073_1.-----------------MYLVIVNYNKD-----IERKRIDYLIEKWSKMG-KIEKIKKTALIVDID--DMDEFIKEISSKLEGDEE-----KKLKIYKIEE--VKKKIPK.78
WP_013099905_1.----------------MKYLIIVDYDRD-----SERKRIDYLIEKWRNKG-KIEKIKKMAILAELE--DIEEFLNEILARLE--RE-----DELKVYKVEE--INNKISP.77
WP_013799800_1.----------------MTYLVVVDYEKD-----SERKRIDYLIERWSNRG-SIEKIRKMAIIVDVD--NIDDFIKDIMSRLEGNPE-----EKVKVYEIKE--VKKTIPS.79
WP_018153917_1.----------------MSNLIVVDYDKD-----AERKRIDYLIERWSNKG-NLEKIRKMAIIADID--DVDGFVKDIMSRLEGNPE-----EKVRVYEIKE--LKKTISS.79
WP_042690065_1.----------------MKYFVVVSYDDD-----AERKRVDYLLSKWAERA-DVEKPRGMTFIIETE--QEKEFFEELFSRIEGNPS-----EKVRIYRVTE--EWPELRK.79
WP_048811157_1.----------------MGYILFVAYDND-----AERKRIDYLLDKWSSRA-TVKKPRGMVFYLETD--DAQGFLEELFSRLEGNAE-----EKVEVYEARE--VRKTVKA.79
WP_014121472_1.----------------MGYILFVAYDND-----AERKRIDYLLDKWSSKA-TVKKPKGMVFYIETD--EAQGFLEELFSRLEGNAE-----EKVEVYEAKE--VRKTVKA.79
WP_014011619_1.----------------MGYIVFVTYDND-----AERKRIDYLLDKWSSRA-TVKKPRGAVFYIETD--DTQEFLEELFSRLEGNAE-----EKVEVYSARR--VEKGVEA.79
WP_014789560_1.----------------MGYIIFVTYDND-----AERKRIDYLLDKWSSRA-TVKKPRGVVFYIETD--EPQEFLEELFSRLEGNAE-----EKVEVYSAKR--VENRVRA.79
WP_050003279_1.----------------MGYIIFVTYDND-----AERKRIDYLLDKWSSQA-TLKKPRGTVFYIETD--NTRDFLEELFSRLEGNAE-----EKVEVYYAKK--VESNVKA.79
WP_088858745_1.----------------MGYIIFVGYETD-----AERKRIDYLLDKWSGKA-VIKKPRGTVIYIETK--NPTEFLEELFSKLEGNVD-----EKVEVYKAEP--LREGVEA.79
WP_010476909_1.----------------MGYIIFVSYETD-----AERKRIDYLLDKWSSRA-VIKKPRGTVVYIEAD--SLTEFLEELFSRLEGDIG-----EKIEVYEARP--LKKAVES.79
WP_048177849_1.----------------MEYLFTIDYGSD-----AERKRIDYTVERWSDRA-KVKKPRGAVLLFKGP--DVDEFIEDLYSRLDIDA------GKVEIFRIEP--YEPSVEK.78
WP_048145794_1.----------------MDYIVFVDYFSD-----AERKRIDYAIERWRDKA-DITREKGIIIRFKDK--NIDSFLDDLYSRLDTGR------EQVRVFEADM--HEPEIRA.78
WP_042699369_1.----------------MDYLIVVDYQSD-----AERKRIDYAIERWKDKG-KIHKPKGTVIHYAGD--DVDEFLDDLYSRLSVGA------GSVHIFAGDT--YSPDINE.78
WP_042699369_1.----------------MDYLIVVDYQSD-----AERKRIDYAIERWKDKG-KIHKPKGTVIHYAGD--DVDEFLDDLYSRLSVGA------GSVHIFAGDT--YSPDINE.78
PKL62238_1.....----------------MGYLLIVDYESD-----AERKRIDYAIERWGDRA-EISKPKGTAMVFKGA--NIDEFLEDLYSRIEGDR------RKVEVYRMEE--YHPEVEE.78
WP_049937822_1.TATNSNHGSSTEDDDRETYLIVLDYTDE-----AERKRVEYLLNNR-DGT-DVSPLRGFVRSVKTD--DLPGLYEDLSAKV---DD----VEDLEVKRLED--VDTTPSE.92
OKY77357_1.....MIEIEKEKKGNKESEEKKYLFVVSYDTD-----AERKRVDYLFNNW-EKG-EIEKPKGLVRLVNTS--NYLNLYKSLTSKI---PE-----KNIDSYEINK--NEPDISP.91
WP_049926276_1.---------MSEDNNEGRYLYIVDYEED-----AERKRAEYLFNNW-DEG-EINRPSGLVRIAEGV--DRDELYEQLVAKV---PE-----QQVDICRLEP--VDTDVEP.82
WP_087715649_1.--MSNTDPSSDETSTDTEYLFVVDYEDD-----AERKRIEYLFNNW-DDG-EIAKPSGLVRVGQSV--SHDELYQDLVAKV---PE-----EQVQSYRLEP--VETDVAP.89
ERH02099_1.....--------MAQNDNNTPRYLFTVEYDEE-----AERKRVEYLFSNW-DSG-QITAPNGLVRIAEGI--DREELHEQLLTKV---PE-----EQVDVYELET--TAMDIET.83
ESS11252_1.....-------------MGSDRYLYAVEYESD-----AERKRVEYLFNNW-ADG-TVDQPDGLVRVAEGA--AHDELYEQLLTKV---PA-----ERVSAYELKP--ADTDVEP.78
WP_012807457_1.------------MSEDGAYLVVVDYEDE-----RERKRAEYLLDNW-EDG-TIESLDGMSRVVRDV--DIDELYDQLAAKV---PE-----EELSAYELNR--VDTEATQ.79
WP_008446014_1.------------MTADDTYLVVVDYEAD-----TERKRAEYLLDNW-DEG-SVESVRGLTRLVSGT--DIHELYDALAAKV---PE-----DQIETYDLTP--VNSEAST.79
WP_026190340_1.------------MSEDGAYLVVVDYEDE-----RERKRAEYLLDNW-EDG-TIESLDGMSRVVRDV--DIDELYDQLAAKV---PE-----EELSAYELNR--VDTEATQ.79
PDB:.2IVY/SSO1404..............MLYLIFYDITDD-----NLRNRVAEFLKKKGLD-----RIQYSVFMGDLNSSRLKDVEAGLKIIGNRKKLQEDERFFILIVPITENQFRERIVI
PDB:.7F84_A/LIC12917...........MFIIVCYDVETITQEGRARLRKVAKTCESHG------QRVQKSVFECQLEPADYLQFEAKLSKIIN------SKTDNLRIYSLDAISVSKIKQF

                   //.....................................TBP/DUF3568.........................................//
WP_015313867_1.ETRKIKFQLNK-SKEAVDSFINYLLAQKKAIYK-NS-IGNTKYYESATRRGYAQIRVN-INETLESVE------VQVEINSEEPNRSHLINFFTEEIELF.166
OIQ05980_1.....GRKKTEMSFDV-DVNAVEKFVGFVMAKRKGVLKRESKKPRIKEYSVYTNKGGGDVVVK-FSSGEDGKGTTKETKLLIVIEAFEPALSYLYRIITEDFEYF.172
PIV28043_1.....GRKKTEMSFDV-DVNAVEKFVGFVMAKRKGVLKRESKKPRIKEYSVYTNKGGGDVVVK-FSSGEDGKGTTKETKLLIVIEAFEPALSYLYRIITEDFEYF.172
PKP60869_1.....GRKKIEMSFNA-DVNVVEKFAGFVMAKRKGVLKRESKKPRIKEYSVYTNKGGGDVAVR-FSGGEEGEG-NKETKLLIVIEAFEPALSYIYRIITEDFEYF.171
ARM76973_1.....IQETKTYLLNK-SLHDTKTFLNFIIAKNKGIYL-GK-TEEADIYDIYTRKGIVRTFVA-LKGDTNKTQ------IILSYEGTKEAVNKIEEEIEKEIKIF.164
WP_048099576_1.YSLKATFSFSK-GISEVKSFVDYLIAKNKGIYL-GKFSGEE--YEIYTRKGLVRLTVN-LSQEG-KVT------LKLEMKGPKDAVDRVFQEISKELEIF.161
WP_011011773_1.KEKVLEYYSKE-SENVVKRLLRYIFSKNNLYYY-SS-DNYSEEYFTLTRKGRVSIEVS-VKPISGGTK------VKLRLSGYGEAVEYVAEKIDNDIRLL.171
WP_056934134_1.ATTVLEYQSND-PPEIVKKLLGYVFSKNNVRYY-SS-DLKSTKYFVLTRKGRVDIEVT-IDSRNDKTW------VKLFLSGYGEAVEHMAKKLKEDLDSL.171
WP_048165623_1.NKVELEYITNE-NPQIVRKLLRYILSKCNAYYV-SS-DDLSMTYGAYTKKGRVEIRIE-IDEKSTNET-----QIVISLVGYGEVVEYMAKKLREELSLL.172
WP_055429724_1.TRREIEYLTRE-EPVVVRKLLKYVLSKFGAYYV-SS-EGNVSRYRAYTKKGRMEVEVL-VEERDNGTA------VVIAIEGYGTAVEDMARKLERELSLL.171
WP_062390972_1.KVVEIRYTSNE-EPAIVAKLLRYVLSKFGAYYV-SG-EGSVSRYRAYTKKGRLEITVS-VEEEDNGTA------VDISIEGYGSAVEDMAKKLRRELSLL.171
WP_048152318_1.KVVEIRHTSSE-EPAIVAKLLRYVLSKFGAYYV-SG-EGSVSRYRAYTKKGRLEITVS-VEEEDGGTA------VNISLEGYGSAVEDMAKKLERELSLL.171
WP_058937978_1.KVVEIRHTSSE-EPAIVAKLLRYVLSKFGAYYV-SG-EGSVSRYRAYTKKGRLEMTVS-VEEEDNGTA------VNISIEGYGSAVEDMAKKLRRELSLL.171
WP_015733073_1.ERKSLKYKIN--DMDFMEKFIDYLMAKMGGAYI-SS-IGKTAKYEIYTKKGKGIIKIT-LESSY----------AIFEIEGYGEVVDYLSERINEEMSFI.163
WP_013099905_1.RKMILSYKLK--DKKALESFVDYLMAKLKASYE-LT-LGNVKKYTAYSKMGNVSILVK-VDNEK----------VIFELEGYGEGVEKIKERIDKDMKLF.162
WP_013799800_1.KKITLNYEVAD-KKEVVEGFLKYLMAKIGASYE-CS-INDTKKYEAYTKKGSCSISIK-LKEKGNKLI------INFEIEGYGESVDLIKNKIDNDMKLF.169
WP_018153917_1.KKTTLSYKITD-KKEAVEGFLKYLMIKLGASYE-CS-VNGTKKYEVYTKKGNCSISIK-LKENNENKL-----IINFEIEGYGESVDLIKNKIDNDMKLF.170
WP_042690065_1.RSATREYLLAE-EEGFVRRFLAYLCSKLGGTF-----SPEEGTCEVYTRKGRGVIQFS-LRGTPRGTV------VRVTVSGYGEVVDYLLSRVDEELKPL.166
WP_048811157_1.RRRRLEYTIDE-EAKVVERFIDYLLSKMNATQV-ES-SGEEKTYSVYTRKGRGTIRLR-VRNGG-RTF------VFFEIEGYGEVVDLLADRIDEEMKLF.168
WP_014121472_1.RKRRLEYTIEE-EPKVVERFLDYLLSKMNATQV-ES-SGEEKTYSVYTRKGRGTIRLR-IQNGG-RTF------VLFEIEGYGDVVDLLADRIDEEMRLF.168
WP_014011619_1.KRRTLEYTIAE-ERKVVERFIDYLLSKMNAGYS-HS-ENEAKVYGVYTRKGRATIRAT-IDGNG-RTR------VTLEIEGYGDAVDFLAERIDEELKLF.168
WP_014789560_1.KRRTLEYTIGE-ERKVVERFIDYLLSKMNAGYS-HS-ENEAKVYSVYTRKGRATIRAT-IEGNG-RTK------VTLEIEGYGDAVDFLAERIDEELKLF.168
WP_050003279_1.RRRVLEYTINE-EKKVVEKFIDYLLSKINSSYS-HS-EDDTKIYNVYTRKGRATIRAT-IHGDRRTR-------TSLEIEGYGDVVDFLAERIDEELKLF.168
WP_088858745_1.RKKRLDYTLPE-EKRIVERFVGYLLSKLNASLT-SS-DAVARIYNVYTRKGRATIKVI-ISGNGKSH-------VTFEIEGFGEAVDFLADRIDEELKIF.168
WP_010476909_1.KKRVLEYTLPE-EKKVVERFIEYLLSKLNASPT-GS-DPIAKSFSVYTRKGRATIKVI-TRGDGSSQ-------VVFEIEGFGEAVDFLAERIDEELKLF.168
WP_048177849_1.QSRKLVYESPE-RFEAIQSFLNYLMSKLGASFE-YS-TESASYYTAYTKKGQAELEIRWIGCAGGDEGEERALRFVISVEGYGNVVDFIADKLDEEMRIF.175
WP_048145794_1.REEVLIYHSSA-GSDVIEKFLGYIMAKINAHYE-FR-QNGIIRYAASTKKGQARIEIS-ISDSEAGSK------TRIKISGYASSAKFLHDRIDEEMKTF.168
WP_042699369_1.QTTQLKYTTTM-DSAAVENFLKYIMNKLGASFE-GT-KQGIKSYTAYTKKGQAGIDVA-LTAGD-NTA------VLVIVRGYGEVVSFIAGRIDKELKVF.167
WP_042699369_1.QTTQLKYTTTM-DSAAVENFLKYIMNKLGASFE-GT-KQGIKSYTAYTKKGQAGIDVA-LTAGD-NTA------VLVIVRGYGEVVSFIAGRIDKELKVF.167
PKL62238_1.....KTRRLRYESKE-EIEVLEKFLSYLMSKINAGYD-YR-TGNTKKYFVTTKKGQASIEIT-LKGNG-TTD------VLITILGYGEIVEFLAEKISEEMEVF.167
WP_049937822_1.TDLEFDIETDV-DADRIEWLFESLMNKRDAAVA----NPGKNVYKLSTRKGTAQCWYR-TTDNPDGSI-----TVTVYTSGYGEAPRVVRDYLKDEMEGF.181
OKY77357_1.....QTISVNKSMKE-DPKALNEFVEYMLSKKNAVIK----NHSKNKYEVYTKKGRAEISYK-VDEEDNKTN------LMIKIEGFSPAPQFLRDFFNKELNYY.179
WP_049926276_1.ESVTVEQRIAA-SIDAVETFVEYILSKKKAVLQ----SAARNEYEVYTKKGRAEVGYR-LNETEGQVV------AQININGYPPAPSFLAEFFEAELTEY.170
WP_087715649_1.ETLTVEQSIDA-PADAVETFVEYIFSKRKAVLQ----SAPHNEYEVYTKKGRADVTYR-LDADDESTD------VTVTITGHAPAPGFLANFFETELKAY.177
ERH02099_1.....EREVIEQEITA-SLDAVETFLDYVFSKKKAVLQ----SSSRNEYEVYTKKGRAEVSYD-LQATGDSVT------VRVRVDGYPPAPSFLAEFFQTELSEY.171
ESS11252_1.....ETVRVSQTVAA-PVEAVESFVQYMFSKKKAGLQ----SAKHNEYEVYTKKGRATVSYT-LTDSGSDVF------VEISVVGHDPAPTFLGDFFETELTNY.166
WP_012807457_1.VQATIDETFDDVEAERVEWAMESIMKKRKAVDQ-GSTTDGESLWAVYTKKGRAEIQYD-IREREQNTV-----RLHVVIDGFGEAPEFLREFIEEEIGYM.172
WP_008446014_1.SDERLDIRTQT-DPERVEWAVESVLNKRKAVED----PEQDDTYAVYTKKGRATVRYD-VVPRDDGST------LEIRIEGYGEATEFLRDFFEEELGYM.167
WP_026190340_1.VQATIDETFDGVEAERVEWAMESIMKKRKAVDQ-GSTTDGESLWAVYTKKGRAEIQYD-IREREQNTV-----RLHVVIDGFGEAPEFLREFIEEEIGYM.172
```

Supplementary Figure 3: Multiple Alignment of arCOG08177 sequences

Cas2 and TBP domains are indicated above the alignment. Alignment was colored using http://www.bioinformatics.org/sms2/color_align_cons.html tool with default parameters for amino acid grouping and consensus 80%. Alignment with two previously studied Cas2 [78,79] is based on HHpred alignments. Positions corresponding to catalytic residues of Cas2 are highlighted by red. A few amino acids from C-terminus of several sequences were truncated.
