# Supplementary material for: Diversity, origin, and evolution of the ESCRT systems
Source: mBio. 2024 Feb 21;15(3):e00335-24. doi: 10.1128/mbio.00335-24 (PMC10936438; doi:10.1128/mbio.00335-24)
Supplement: Figure S4 — Alignment and structural models for CdvA helical hairpin and Steadiness box (SB). [file mbio.00335-24-s0004.pdf]

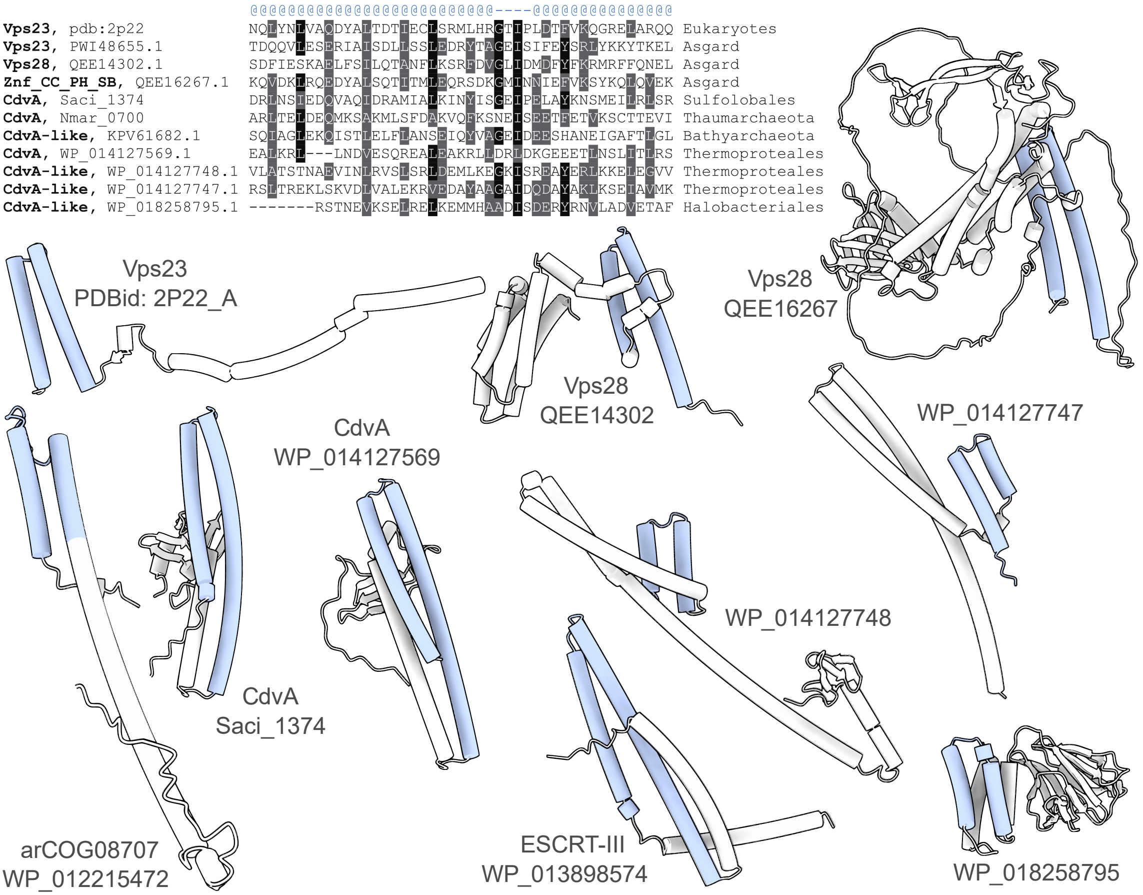

Supplementary Figure 4: Alignment and structural models for CdvA helical hairpin and Steadiness box (SB).
Manual alignment of SB-like alpha helix hairpin is based on the respective HHpred alignments with profile PF18822.4 corresponding to CdvA. Protein accession, locus tag, gene name or PDB ID (for eukaryotes) are indicated on the left. Major taxonomic lineages are indicated for each sequence on the right. Details for the respective HHpred searches are provided in the Supplementary Table 4. Alpha helices are denoted by “@” above the alignment. Alignment coloring is based on default amino acid groups implemented in http://www.bioinformatics.org/sms2/color_align_cons.html tool with 50 % consensus. Structural models of selected proteins containing SB or CdvA are shown. The SB-like hairpin is shown in blue. ESCRT III protein, which also has a distinctive alpha helix hairpin is shown for comparison.
