# Supplementary material for: Diversity, origin, and evolution of the ESCRT systems
Source: mBio. 2024 Feb 21;15(3):e00335-24. doi: 10.1128/mbio.00335-24 (PMC10936438; doi:10.1128/mbio.00335-24)
Supplement: Figure S6 — Structural models for the Meth_adaptin clade. [file mbio.00335-24-s0006.pdf]

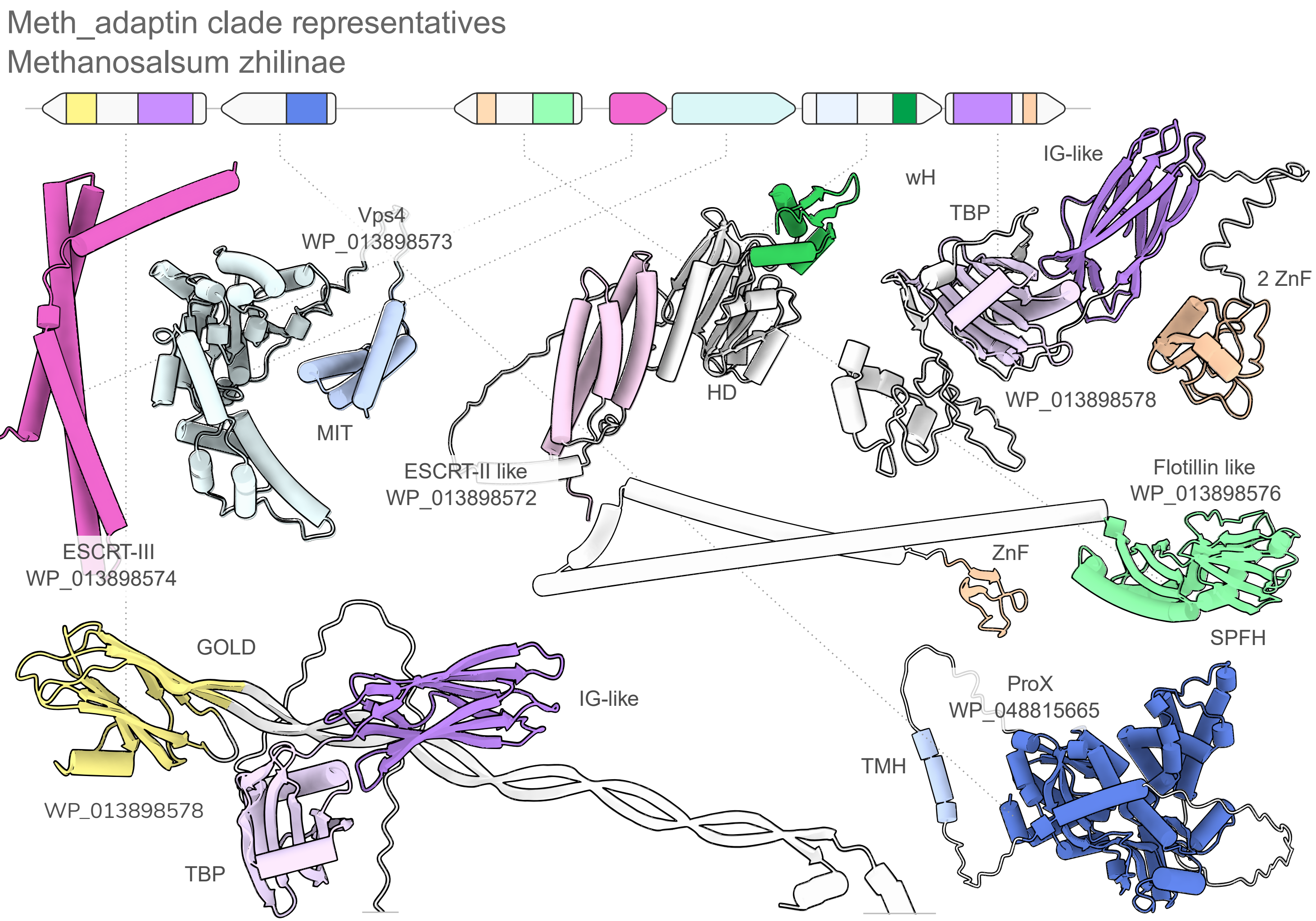


**Supplementary Figure 6:** Structural models for the Meth_adaptin clade
The gallery of structure predictions obtained for the Meth_adaptin clade (Figure 3A) using the gene neighborhood from Methanosalsum zhilinae as representatives is shown (Supplementary table 3). The gene neighborhood organization is shown on top. Proteins are colored by common structural domains found in the ESCRT gene neighborhoods. Protein names are assigned by sequence or structural similarity. Unstructured termini and long linkers are hidden. Abbreviation are as per previous legends with addition of: HD, Helical Domain; TMH, Transmembrane helix.
