# Supplementary material for: Diversity, origin, and evolution of the ESCRT systems
Source: mBio. 2024 Feb 21;15(3):e00335-24. doi: 10.1128/mbio.00335-24 (PMC10936438; doi:10.1128/mbio.00335-24)
Supplement: Figure S7 — Structural models for the Halo_adaptin clade. [file mbio.00335-24-s0007.pdf]

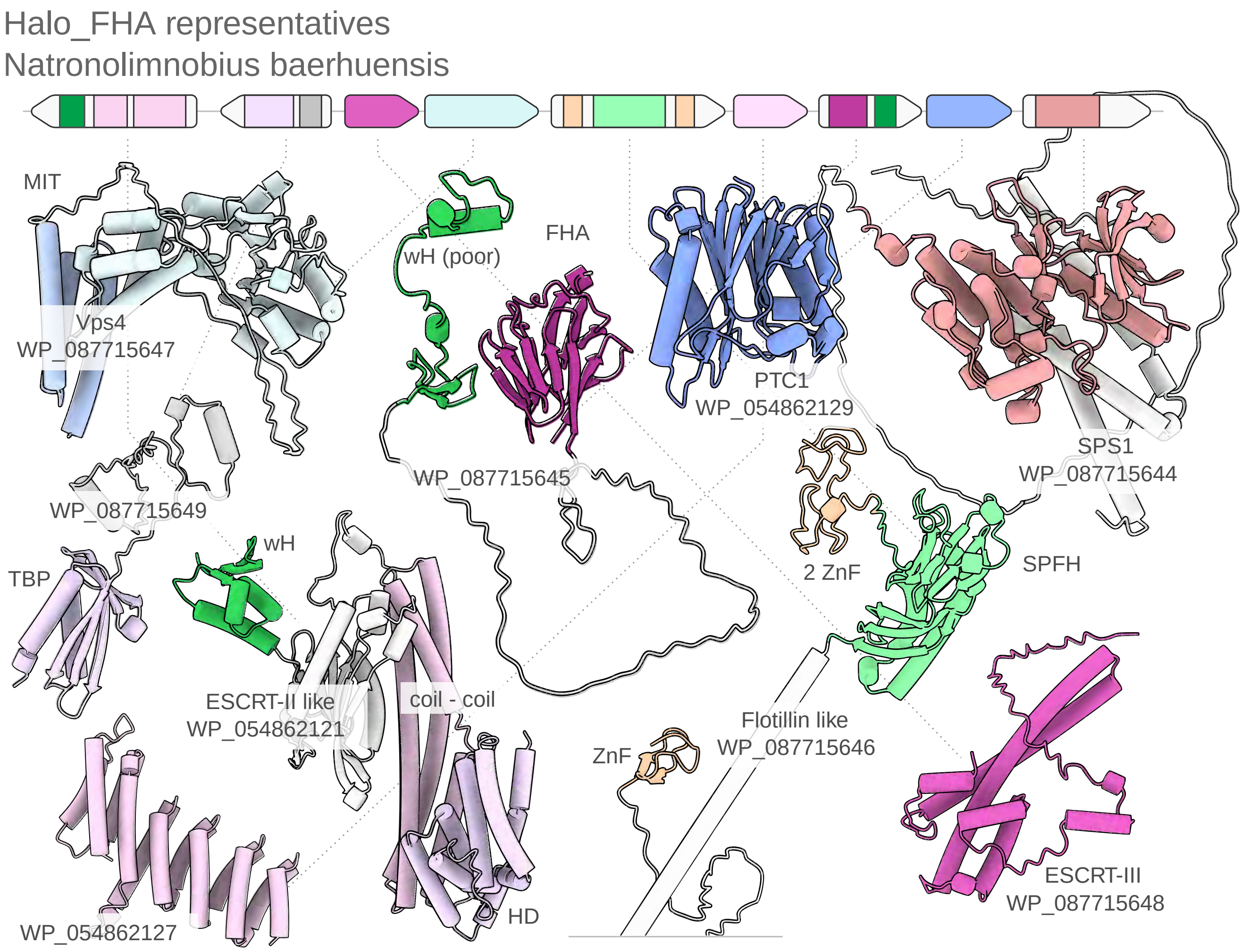

Supplementary Figure 7: Structural models for the Halo_adaptin clade
The gallery of structure predictions obtained for the Halo_adaptin clade (Figure 3A) using the gene neighborhood from Haloquadratum walsbyi as representatives is shown (Supplementary Table 3). The gene neighborhood organization is shown on top. Proteins are colored by common structural domains found in the ESCRT gene neighborhoods. Proteins names are assigned by sequence or structural similarity. Unstructured termini and long linkers hidden. Abbreviation are as per previous legends with the addition of HD, Helical Domain; TMH, Transmembrane helix.
